# Keeping all secondary structures of the non-coding region in the circular genome of human bocavirus 2 is important for DNA replication and virus assembly, as revealed by three hetero-recombinant genomic clones

**DOI:** 10.1080/22221751.2019.1682949

**Published:** 2019-11-01

**Authors:** Linqing Zhao, Tao Wang, Yuan Qian, Jingdong Song, Runan Zhu, Liying Liu, Liping Jia, Huijin Dong

**Affiliations:** a Laboratory of Virology, Beijing Key Laboratory of Etiology of Viral Diseases in Children, Capital Institute of Pediatrics, Beijing, People’s Republic of China; b State Key Laboratory for Infectious Disease Prevention and Control, National Institute for Viral Disease Control and Prevention, Chinese Center for Disease Control and Prevention, Beijing, People’s Republic of China

**Keywords:** Human bocavirus 2, circular genome, genomic clones, DNA replication, virus assembly

## Abstract

The episomal structures of all human bocavirus (HBoV) genotypes have been deciphered, including the circular genome of HBoV2 (HBoV2-C1). To discern the role of the circular HBoV2 genome, three distinct linearized HBoV2-C1 genomes were cloned into pBlueScript SKII(+) to obtain pBlueScript HBoV2 5043–5042 (retaining all secondary structures), pBlueScript-HBoV2 5075–5074 (retaining hairpin number 2 and the 5′ terminal structure), and pBlueScript-HBoV2 5220–5219 (retaining only the 5′ terminal structure at the 5′ -genome end). The recombinant plasmids were separately transfected HEK293 cells, revealing that more HBoV2 DNA had accumulated in the pBlueScript HBoV2 5043–5042-transfected HEK293 cells at 72 h post-transfection, as determined by real-time PCR. However, more mRNA was transcribed by pBlueScript-HBoV2 5075–5074 than by the other constructs, as determined by dot-blot hybridization and RNAscope. No significant differences in NS1-70 protein expression were observed among the three HBoV2 genomic clones. However, electron microscopy showed that HBoV2 virus particles were only present in the pBlueScript HBoV2 5043–5042-transfected HEK293 cells. By using three hetero-recombinant HBoV2 genomic clones in HEK293 transfected cells, only the genome with intact secondary structures produced virus particles, suggesting that retaining these structures in a circular genome is important for HBoV2 DNA replication and virus assembly.

Human bocavirus (HBoV) was first identified as an emerging virus and a possible pathogen responsible for acute respiratory infections in paediatric patients in 2005. By 2010, four genotypes (HBoV1–HBoV4) had been reported, of which HBoV1 was thought to be a respiratory pathogen capable of causing severe and even life-threatening respiratory disease in infants and young children. The roles of the other genotypes (HBoV 2–4) in human diseases (most isolates were found in faecal samples) remain uncertain [1–4]. However, according to the principles of Koch’s modified postulates, HBoVs cannot be confirmed as the causative agents of disease without a workable animal disease model and/or a highly efficient virus cultivation system, both of which remain a major challenge [5–7]. Therefore, studying the emerging paediatric HBoV pathogens at the molecular and structural level is an alternative strategy for revealing the replication mechanism, which could be targeted by therapeutics and vaccines [8].

HBoVs are small, icosahedral, non-enveloped viruses (18–26 nm in diameter) containing a single linear, negative- or positive-sense, single-stranded DNA molecule [9]. The length of the linear single-stranded HBoV genome is only about 5 kilo base pairs (bp), excluding the terminal 32–52 nucleotide (nt) sequences that play a key role in viral replication [4]. According to the new taxonomy system, HBoV1-4, along with gorilla bocavirus, belong to the *Bocaparvovirus* genus, in which HBoV1 and HBoV3 are members of the *Primate bocaparvovirus 1* species, whereas HBoV2 and HBoV4 are *Primate bocaparvovirus 2* species members [9]. The replication mechanism used by HBoVs remains elusive, with two conflicting models proposed: the rolling hairpin [10,11] and the rolling-cycle [12]. Replication in other parvoviruses occurs via the rolling-hairpin model, with generation of concatemer intermediates characterized by a head-to-head or tail-to-tail structure. However, replication in *bocaparvovirus* species may differ from that of other parvoviruses with the discovery of head-to-tail structures in all HBoV genotypes, and no head-to-head or tail-to-tail intermediates identified [13–16]. The HBoV2-C1 circular genome was detected in a clinical specimen in our laboratory [16]. Its complete circular genome (HBoV2-C1, BJQ435: GenBank Accession No. JX257046) is 5307 nucleotide (nt) long with four open reading frames: NS1 (1923 nt, position 256–2178), NP1 (648 nt, position 2405–3052); VP1 (2004 nt, position 3039–5042) and VP2 (1617 nt, position 3426–5042), plus a 520 nt-long terminal non-coding region (NCR) (at nt positions 1–255 and 5043–5307) containing secondary structures, hairpins 1 and 2 (with a specific rabbit ear-like structure) and a 5′ terminal structure. The secondary structures of the episomal forms of the NCRs have been determined for all HBoV genotypes, and for a canine bocavirus. Conserved secondary structures in episomal NCRs are likely to play an important role in bocavirus replication [15]. Future research efforts delving deeper into the replication mechanism of HBoVs are likely to improve our understanding of the pathogenetics of HBoVs [14].

Therefore, in the present study, to evaluate the role of the circular genome in HBoV replication, we designed amplification primers targeting the conserved secondary structures in episomal NCRs from HBoV2-C1 to obtain three distinct linearized genomic HBoV2-C1s containing different secondary structures in the NCRs of the 5′ ends of their genomes. These amplified linearized genomic DNAs (gDNAs) were cloned into the following plasmids: pBlueScript SKII(+) to obtain pBlueScript HBoV2 5043–5042, keeping all of the natural secondary structures, pBlueScript-HBoV2 5075-5074, keeping the two hairpins and the 5′ terminal structure, and pBlueScript-HBoV2 5220–5219, keeping only the 5′ terminal structure at the 5′ end of the genome, each of which were separately transfected HEK293 cells. The production efficiency of HBoV2 DNA, RNA, proteins and viral particles from the three genomic clones was evaluated.

## Materials and methods

### Cell culture

The human embryonic kidney 293 (HEK293) cell line obtained from the Cell Resource Center (Institute of Basic Medical Sciences, Chinese Academy of Medical Sciences & Peking Union Medical College) was cultured in Dulbecco’s Modified Eagle Medium (DMEM) supplemented with 10% foetal bovine serum (FBS), penicillin (100 U/ml) and streptomycin (100 mg/ml).

### Viral DNA extraction

The faecal sample (BJQ435) used in this study was obtained from a child with acute gastroenteritis in Beijing, China, and was confirmed to contain a circular HBoV2 genome with a head-to-tail sequence (HBoV2-C1) [16]. Nucleic acids (DNA and RNA) were extracted from 150 µl of specimen BJQ435 using the QIAamp MinElute Virus Spin Kit (Qiagen GmbH, Germany) according to the manufacturer’s instructions.

### Amplification of HBoV2 genomic fragments and construction of a full-length HBoV2 clone

To obtain the complete genomic sequence of HBoV2, including the terminal region [16], primers were designed according to the genomic sequence restriction map for HBoV2-C1 (BJQ435: GenBank Accession No. JX257046), with unique restriction endonuclease cleavage sites at nt 666 (*Avr*II), nt 2205 (*Xba*I), nt 3498 (*Age*I) and nt 4273 (*Eco*RI) (Table 1). High-fidelity long PCRs were performed using Platinum® Taq DNA Polymerase (Invitrogen Life Technologies, USA), and the resulting PCR products were purified, cleaved and ligated to obtain sequences exceeding the whole genomic sequence of HBoV2-C1, followed by cloning into plasmid pBlueScript SKII(+) using a DNA ligation kit (Promega, Madison, WI. USA). Recombinant DNAs were separately transformed into *Escherichia coli* DH5α to obtain purified pBlueScript-HBoV2 plasmid DNA for fragments 1–4.

**Table 1.** Primers used to construct recombinant plasmids for the full-length HBoV2 genomic clones (fragments 1–4) in pBlueScript-HBoV2 in this study.

Primers	Sequences (5'-3')	Position in HBoV2-C1	Length(bp)	fragments
HBoV2-4F (EcoRI)	atacatcagacagcacgg	4232–4249	1865	4
HBoV2-4R-BamHI (AvrII)	ata*gga*tcccagttaccagtgtgccct	806–789		
HBoV2-1F-XhoI (AvrII)	cgc*ctc*gaggggcagaaacagagaaca	526–543	1773	1
HBoV2-1R (XbaI)	tggacgattatccacgtt	2298–2281		
HBoV2-2F (XbaI)	ggcggctatattcctcat	2086–2103	1467	2
HBoV2-2R (AgeI)	agccgcctgtggatatac	3552–3535		
HBoV2-3F (AgeI)	cctaaaccaggcacatca	3405–3422	941	3
HBoV2-3R (EcoRI)	ggtcctactctttgtgcg	4345–4328		

### Construction of HBoV2 genomic recombinant plasmids

Primers were designed against the episomal NCR sequences from HBoV2-C1 (Table 2) to construct the HBoV2 genomic clones. High-fidelity long PCRs were performed using *Platinum*® *Taq* DNA Polymerase (Invitrogen Life Technologies) using plasmid DNA pBlueScript-HBoV2 fragments 1–4 as templates, after which the PCR products were purified, cleaved, ligated and cloned into plasmid pBlueScript SKII (+) to obtain HBoV2 recombinant plasmid DNAs.

**Table 2.** Primers used to construct HBoV2 genomic recombinant plasmid DNAs.

Primers	Sequence (5'-3’)	Position in HBoV2-C1	Length (bp)
HBoV2-5043F-XhoI	ata*ctc*gagtctcttaagcccgttc	5043–5058	1071
HBoV2-4R (AvrII)	ataggatccagtgtgccct	806–789	
HBoV2-4F (EcoRI)	atacatcagacagcacgg	4232–4249	811
HBoV2-5042R-BamHI	ctc*gga*tccttacaacactttattgatg	5042–5024	
HBoV2-4F (EcoRI)	atacatcagacagcacgg	4232–4249	988
HBoV2-5219R-BamHI	cgc*gga*tccgcaaaacagctcctcc	5219–5204	
HBoV2-5220F-XhoI	taa*ctc*gagttacgcaatcgcgaaat	5220–5236	886
HBoV2-4R (AvrII)	ataggatccagtgtgccct	806–789	
HBoV2-5075F-XhoI	tcg*ctc*gagagttcctctccaatgg	5075–5090	1039
HBoV2-4R (AvrII)	ataggatccagtgtgccct	806–789	
HBoV2-4F (EcoRI)	atacatcagacagcacgg	4232–4249	843
HBoV2-5074R-BamHI	cgc*gga*tcctataagcataagcaatg	5074–5058	

### Transfecting HBoV2 genomic recombinant plasmids into HEK293 cells

HEK293 cells grown in 24-well plates were transfected with 1.5 µg of recombinant pBlueScript plasmid DNAs, using the empty pBlueScript vector or lipofectamine reagent as the controls. Lipofectamine® 3000, P3000^TM^ reagents and Opti-MEM® (Invitrogen Life Technologies) were used according to the protocol manual. At 24, 48 and 72 h post-transfection, the HEK293 cells were harvested and centrifuged at 4000 rpm for 5 min, respectively, with supernatants transferred to new Eppendorf tubes labelled “supernatant”, and pellets re-suspended in phosphate-buffered saline (PBS) in tubes labelled “cells” and partly spotted onto acetone-cleaned slides. Nucleic acids were extracted using Trizol® reagent (Invitrogen Life Technologies) from each 125 µl sample labelled as “cell” or “supernatant”.

### Real-time polymerase chain reaction (rPCR) assays

DNAs extracted from the samples labelled “cell” or “supernatant” were tested for HBoV2 using rPCR assays with HBoV2F (5′-TTGCTCCTGGGACTGAACGT-3′) and HBoV2R (5′-TTCCCTGACAGGATCATCTTC-3′) primers, probe HBoV2P (5′-FAM-TCATGATCCAACTAAGAAACTGCGCACCA-BHQ1-3′) and the TaqMan® universal PCR master reagent kit (Applied Biosystems) [17]. Each 25 µl reaction volume contained 800 nM of each primer, 200 nM of each probe, the TaqMan universal PCR Master Mix Reagent components, and 2 µl of DNA. The thermal cycling programme comprised 50°C for 2 min, a 95°C for 10 min, and 40 cycles of 95°C for 15 s followed by 60°C for 1 min. Each sample was considered positive when its amplification plot showed a definite exponential increase in the fluorescent signal.

The purified plasmid DNA pBlueScript-HBoV2 fragments 1–4 were used separately as control DNAs for the standard amplification curve assessing the relationship between the copies/μl of DNA and the cycle threshold value (Ct) of the rPCR.

### Dot-blot hybridization

The digoxigenin-labelled probe was PCR-harvested using Digoxigenin-11-dUTP (Roche, Germany), with dNTPs, HBoV2F and HBoV2R primers and plasmid DNA pBluscrip-HBoV2 fragments 1–4 as templates. Total RNAs extracted from each sample labelled “cell” were DNAse I digested (final concentration, 90 U/ml) for 15 min at room temperature (RT) to remove residual DNA, and each RNA sample (10 µl/dot) was spotted onto a nylon membrane and hybridized to HRP-anti-DIG for dot-blot hybridization according to the protocol manual (Roche, Germany). The results were visualized by reaction with NBT/BCIP.

### RNA in situ hybridization (ISH) technology, RNAscope

An ultrasensitive RNA ISH technology, RNAscope® Reagent Kit (Advanced Cell Diagnostics, CA, USA), employing a unique probe design strategy allowing target-specific signal amplification of RNA by ISH, was developed to detect HBoV2 mRNA transcripts through standard microscopy techniques using 20 ZZ probe pairs (50 bp/pair) designed according to the HBoV2 VP2-coding region’s sequence. The RNAscope analysis was performed using the manufacturer’s procedure. Briefly, the cell-spotted slides were incubated in Pretreatment 1 solution (RT, 10 min), rinsed twice in TBS, and then treated with Pretreatment 3 (diluted 1:15 in TBS; RT, 5 min). After Pretreatment 3, the cells were rinsed twice with TBS and then hybridized to the probes to detect single HBoV2 mRNA transcripts. Microscopic signals were developed by adding DAB-A and -B followed by counterstaining with Mayer’s haematoxylin.

### Immunofluorescence confocal microscopy

HEK293 cells were grown on glass coverslips before transfection with the aforementioned plasmids. At 72 h post-transfection, the cells were fixed with 4% paraformaldehyde (Sigma-Aldrich, 16005) in PBS for 15 min and then permeabilized using 0.3% Triton X-100 in PBS containing 3% bovine serum albumin (Sigma-Aldrich, A7906) for 30 min. After washing three times with PBS, the cells were incubated with rabbit anti-HBoV2 NS1-70 antisera (1:200) (Jiaxuan Biotech, China) at 4°C overnight, followed by incubation with TRITC-conjugated goat anti–rabbit antibodies for 1 h at RT followed by DAPI (4’, 6-diamidino- 2-phenylindole, Life Technologies, D1306) nuclear staining. Images were captured and analyzed by TCS SP8 laser-scanning confocal microscopy and LAS AF software (Leica Microsystems, CMS GmbH, Wetzlar, Germany). To ensure that the rabbit anti-HBoV2 NS1-70 antibodies worked well, the HEK293 cells transfected with the HBoV2 NS1-70-GFP plasmid used as the control were fixed and stained with rabbit anti-HBoV2 NS1-70 at 72 h post-transfection followed by DAPI nuclear staining.

### Western blotting

HEK293 cells were transfected with pBlueScript vector, or the individual HBoV2 genomic recombinant plasmids. At 72 h post-transfection, the cells were lysed on ice for 30 min in lysis buffer (25 mM Tris-Cl, 150 mM NaCl, 1 mM EDTA, 1% NP-40 or 1% Triton X-100, pH 7.4) supplemented with a protease inhibitor cocktail (Roche, 04693132001). The cell lysates were centrifuged at 12,000 rpm at 4°C for 15 min to remove insoluble material. Aliquots (100 μg) of the total protein contents separated by 12% sodium dodecyl sulphate polyacrylamide gel electrophoresis were transferred to nitrocellulose membranes (Pall, 66485). After blocking with 5% nonfat dry milk solution in PBS (RT, 1 h), the membranes were incubated with rabbit anti-HBoV2 NS1 (1:1000) (Jiaxuan Biotech, China) at 4°C overnight. The following day, the membranes were washed three times in PBST and incubated with HRP-conjugated goat anti-rabbit IgG antibody (Proteintech, 1:4000) at RT for 2 h. The bands were visualized by ECL with SuperSignal West Pico PLUS Chemiluminescent Substrate (Thermo Fisher Scientific, USA).

### Electron microscopic screening for virus particles

HEK293 cells separately transfected with each HBoV2 genomic recombinant plasmid or pBlueScript vector were harvested at 72 h post-transfection and ultra-centrifuged at 100,000 ×g for 10 min. The pellets were re-suspended using drops of PBS solution and absorbed on formvar and carbon film coated grids for at least 1 min, and the grids were negatively stained with 1% (W/V) phosphotungstic acid (pH 6.8) for 1 min. After air drying, the grids were observed under a Tecnai TF20 transmission electron microscope (FEI) and photographed by a CCD camera (Gatan, USA) as described by Zhao, et al. [17]

## Results

### Cloning the full-length HBoV2 genome

PCR products from HBoV2 1, 2, 3 and 4 amplified fragments using primers designed according to the restriction maps of HBoV2-C1 were digested, purified, ligated and then cloned into pBlueScript SKII(+) following the cloning scheme shown in Supplementary Materials S1 to obtain plasmid pBlueScript-HBoV2 fragments 1–4, each of which contains either HBoV2 1, 2, 3 or 4 with 100% identity to the corresponding region in HBoV2-C1.

### Construction of the genomic recombinant plasmids

**Figure 1.**
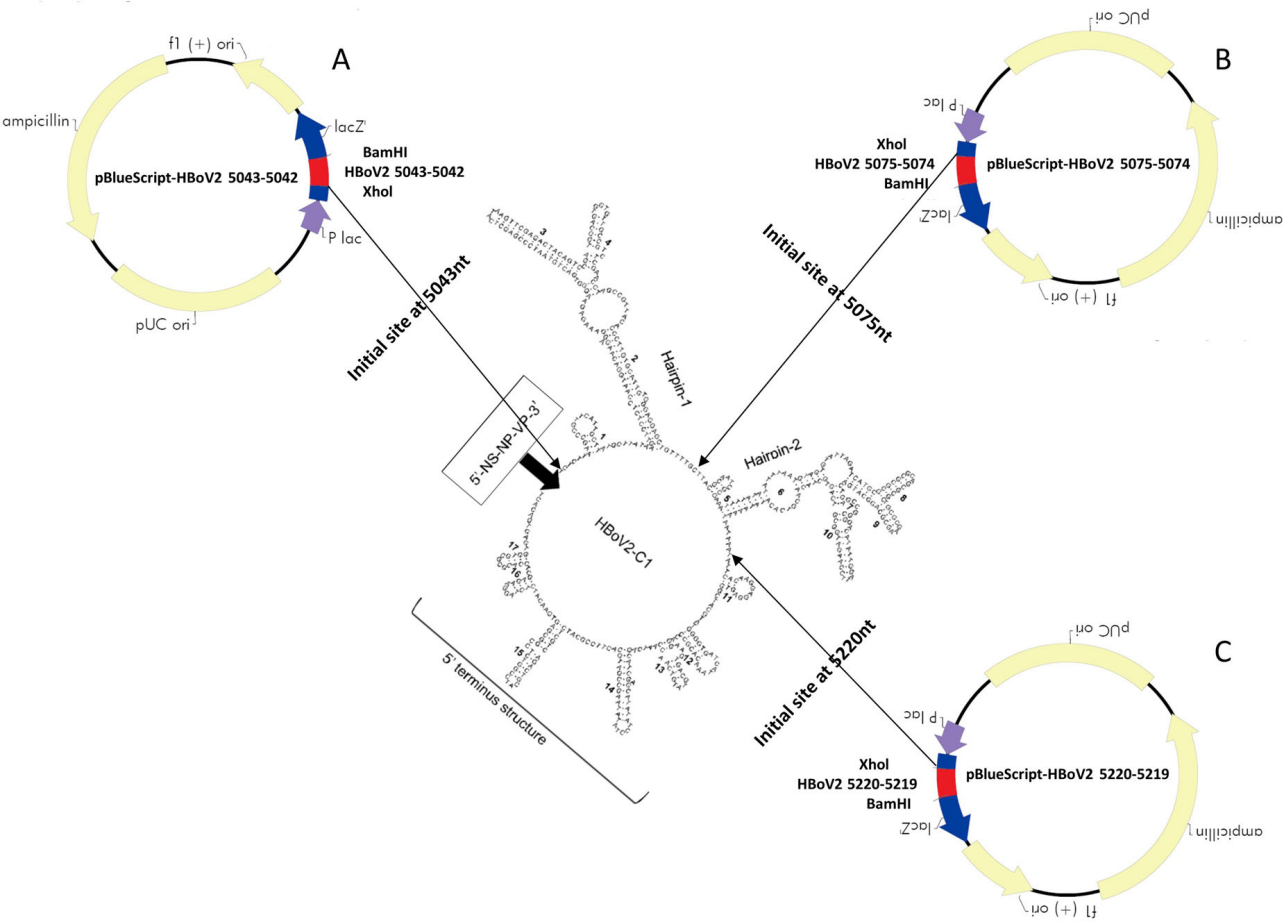
Three HBoV2 recombinant genomic plasmids containing different initiation sites. A: pBlueScript-HBoV2 5043–5042, where the initiation and termination positions of the inserted HBoV2 genomic fragments are at nt 5043 and nt 5042 in vector pBlueScript SKII(+). B: pBlueScript-HBoV2 5075–5074, where the initiation and termination positions of the inserted HBoV2 genomic fragments are at nt 5075 and nt 5074 in vector pBlueScript SKII(+). C: pBlueScript-HBoV2 5220–5219, where the initiation and termination positions of the inserted HBoV2 genomic fragments are at nt 5220 and nt 5219 in vector pBlueScript SKII(+).

Based on the sequences of the conserved rabbit ear-like secondary structures and 5′ terminal structure in the episomal NCR of HBoV2-C1 [16], including hairpins 1 and 2 (Supplementary material S2-A and B), we designed primers HBoV2 5043F-*Xho*I + HBoV2 4R (*Avr*II) to amplify one fragment with an initial site at nt 5043 in HBoV-C1, which was cloned into the 5′ end of pBlueScript-HBoV2 1–4, and primers HBoV2 4F (*Eco*RI)+HBoV2 5042R-*Bam*HI to amplify another fragment with the 3′ site at nt 5042 in HBoV-C1, which was cloned into the 3′ end of pBlueScript-HBoV2 1–4. We then created a new recombinant plasmid, pBlueScript-HBoV2 5043–5042 (Figure 1A), which retained all the secondary structures of the episomal NCR in HBoV2-C1 at the 5′ end. Additional primers were designed to obtain the other two recombinant plasmids: plasmid pBlueScript-HBoV2 5075–5074 with the initial site at nt 5075 and end site at nt 5074 in HBoV-C1 (retaining hairpin 2 and the 5′ terminal structure at its 5′ end) (Figure 1B); and plasmid pBlueScript-HBoV2 5220–5219 with the initial site at nt 5220 and end site at nt 5219 in HBoV-C1 (retaining only the 5′ terminal structure at its 5′ end) (Figure 1C). Each plasmid shared 100% sequence identity with the corresponding region in HBoV2-C1.

### Real-time PCR assays for DNA analysis

**Figure 2.**
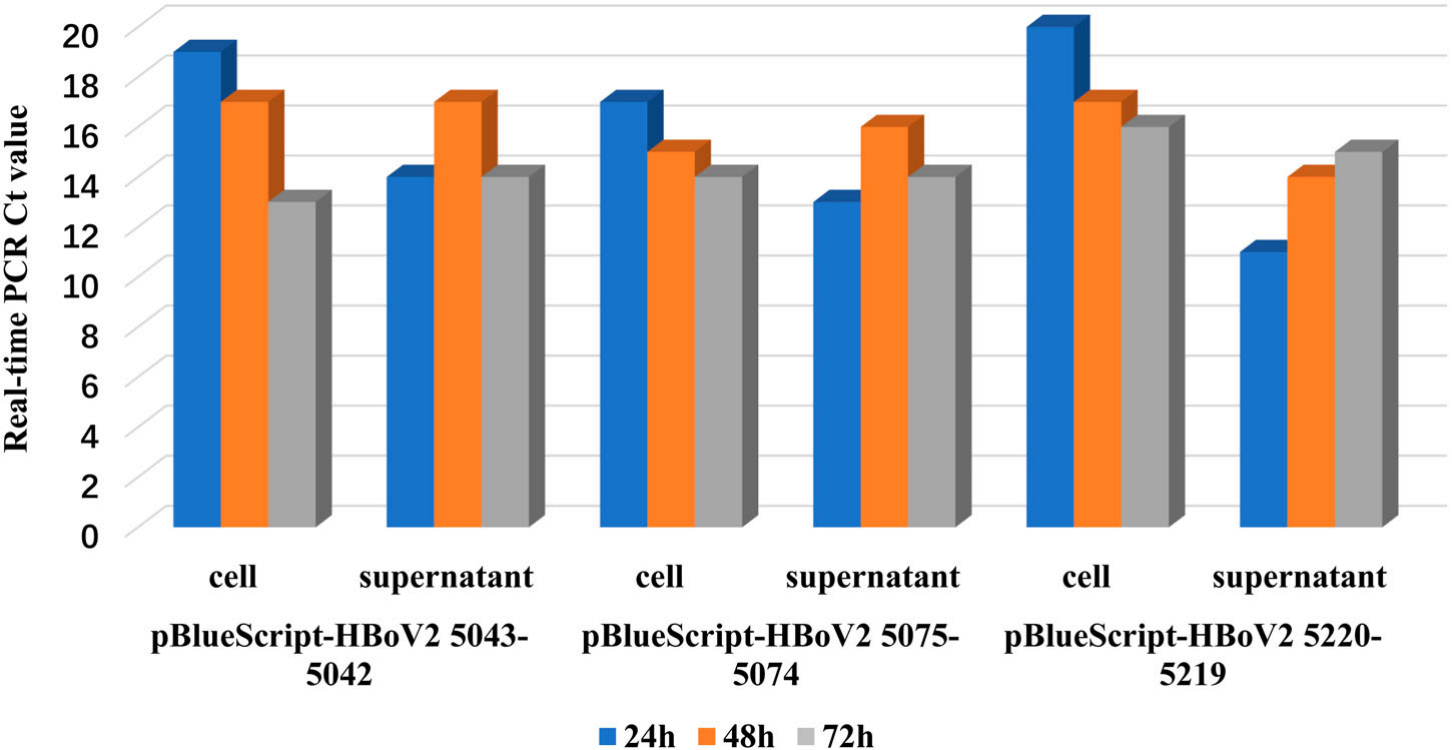
rPCR results for HBoV2 using DNA from the “cell” or “supernatant” of HK293 cells transfected with one of three hetero-HBoV2 genomic recombinant plasmids. 24 h (blue columns), 48 h (orange columns), 72 h (grey columns) denote 24, 48 and 72 h post-transfection, respectively.

According to the rPCR standard amplification curve, assessing the relationship between the copies/μl of DNA and the Ct values using the purified plasmid pBlueScript-HBoV2 DNA fragments 1–4 as the control DNA, the Ct values of the HBoV PCR products in the HEK293 “cells” groups transfected with recombinant plasmid pBlueScript-HBoV2 5043–5042 decreased significantly (*F* = 440.06, *p *= 0.000) over the 24 h (Ct = 19 ± 0.08), 48 h (Ct = 17 ± 0.30) and 72 h (Ct = 13 ± 0.41) post-transfection (Figure 2), indicating that replication of HBoV2 increased the DNA levels in the HEK293 cells from 7.5 ± 0.61 × 10^5^ copies/µl, 2.5 ± 0.35 × 10^6^ copies/µl to 8.5 ± 0.87 × 10^7^ copies/µl, respectively. The Ct values also decreased to some degree in the cells transfected with pBlueScript-HBoV2 5075–5074 (*F* = 1241.80, *p *= 0.000) and pBlueScript-HBoV2 5220–5219 (*F* = 1560.20, *p *= 0.000). The lowest Ct values in the “cell” groups at 24 h (Ct = 17 ± 0.20) and 48 h (Ct = 15 ± 0.11) post-transfection occurred in the pBlueScript-HBoV2 5075–5074-transfected HEK293 cells at 72 h (Ct = 13 ± 0.41) post-transfection and in the cells transfected with pBlueScript-HBoV2 5043–5042. The Ct values decreased more rapidly in cells transfected with pBlueScript-HBoV2 5220–5219 from 24 h (Ct = 20 ± 0.05) to 48 h (Ct = 17 ± 0.11) post-transfection, and in cells transfected with pBlueScript-HBoV2 5043–5042 from 48 h (Ct = 17 ± 0.30) to 72 h (Ct = 13 ± 0.41) post-transfection.

In the “supernatant” groups, the lowest Ct values were recorded at 24 h post-transfection, increasing at 48 h for all the HEK293 cells transfected with any of the three recombinant genomic plasmids. The Ct values decreased at 72 h in HEK293 cells transfected with pBlueScript-HBoV2 5043–5042 and pBlueScript-HBoV2 5075–5074.

### Dot-blot hybridization and RNA ISH RNAscope for RNA analyses

**Figure 3.**
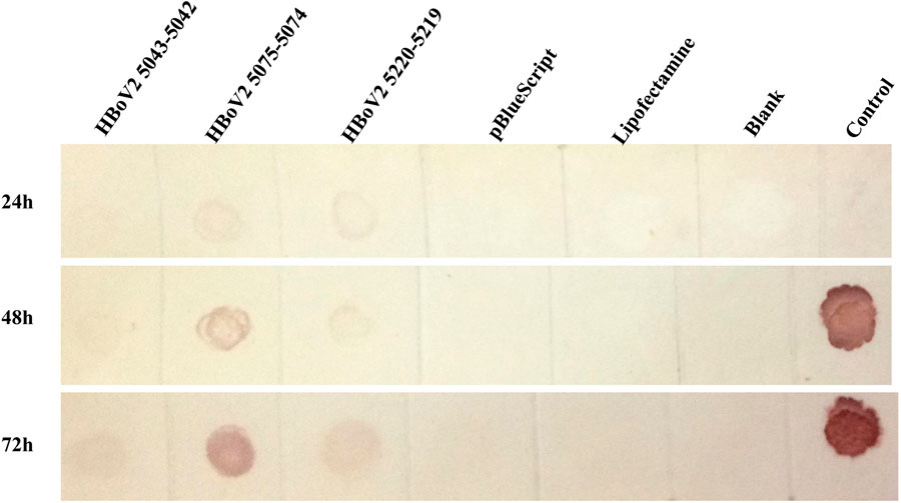
Dot-blot hybridization for HBoV2 using RNAs from the “cell” of HK293 cells transfected with one of three HBoV2 genomic recombinant plasmids (1: pBlueScript-HBoV2 5043–5042; 2: pBlueScript-HBoV2 5075–5074; 3: pBlueScript-HBoV2 5220–5219; 4: pBlueScript vector; 5: lipofectamine reagent; 6: HEK293 cells; 7: negative control (at 24 h) or positive control (DNA from pBlueScript-HBoV2 fragments 1–4, which were not digestible by DNAse I treatment, at 48 h and 72 h)). 24, 48, and 72 h denote 24, 48 and 72 h post-transfection, respectively.

In the dot-blot hybridizations (Figure 3), RNAs from pBlueScript vector, lipofectamine reagent or HEK293 cells were negative, whereas those from recombinant pBlueScript-HBoV2 5043–5042, pBlueScript-HBoV2 5075–5074 and pBlueScript-HBoV2 5220–5219 transfected cells were detectable, increasing time-dependently at 24, 48 and 72 h post-transfection. The highest RNA quantities were observed in pBlueScript-HBoV2 5075–5074-transfected cells.

**Figure 4.**
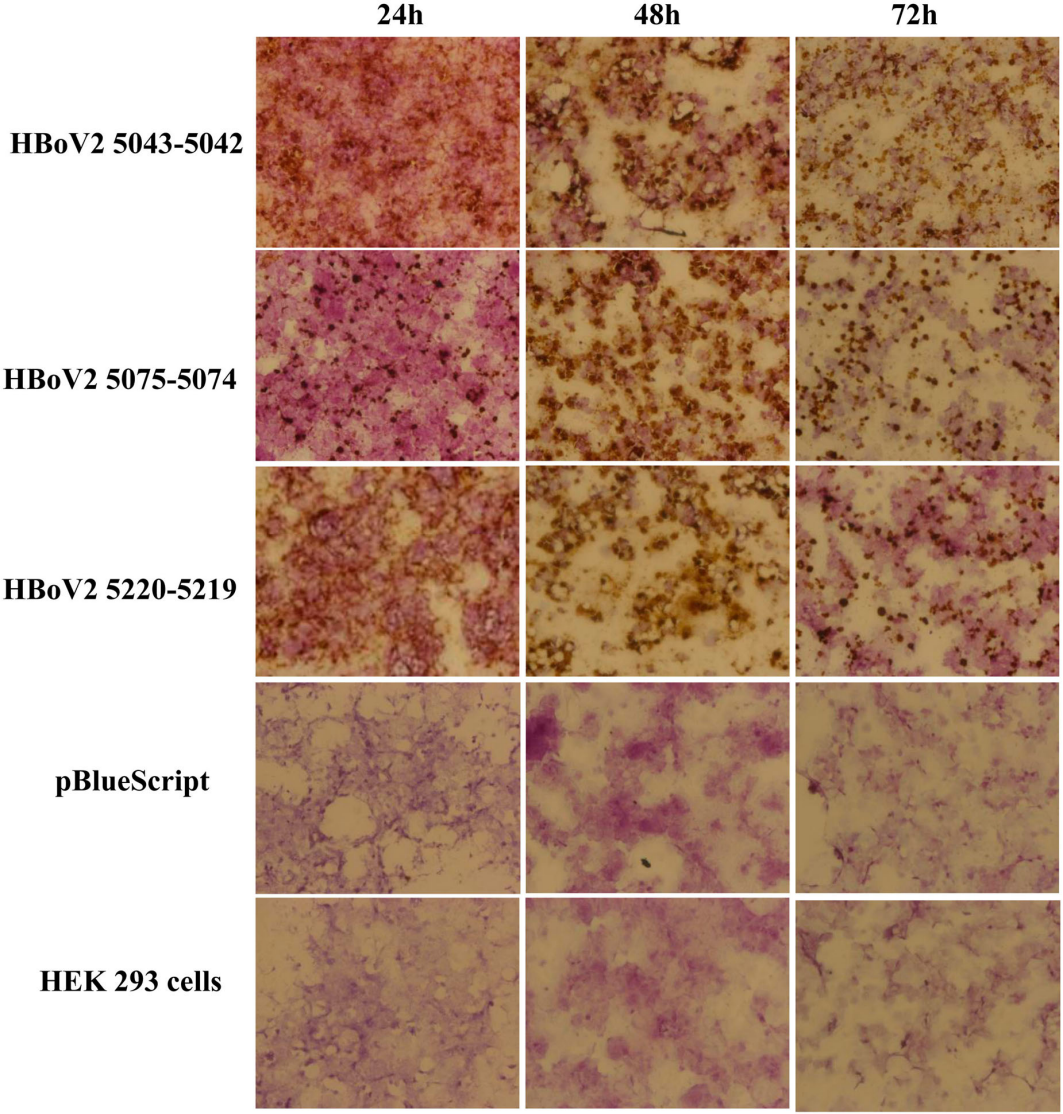
ISH staining of hetero-HBoV2 mRNA transcripts. A: HEK293 cells transfected with plasmid pBlueScript-HBoV2 5043–5042; B: HEK293 cells transfected with plasmid pBlueScript-HBoV2 5075–5074; C: HEK293 cells transfected with plasmid pBlueScript-HBoV2 5220–5219; D: HEK293 cells transfected with pBlueScript; E: Untreated HEK293 cell control. 24 h, 48 h, 72 h denote 24, 48 and 72 h post-transfection, respectively.

In the RNAscope assays, multiple dot-like nuclear signals were seen with the HBoV2 recombinant genomic plasmids pBlueScript-HBoV2 5043–5042, pBlueScript-HBoV2 5075–5074 and pBlueScript-HBoV2 5220–5219, increasing across the post-transfection period of 24, 48 and 72 h (Figure 4). The HBoV2 mRNA signals from the transcripts shown in Figure 6 might indicate the productive phase of the HBoV2 genome.

**Figure 5.**
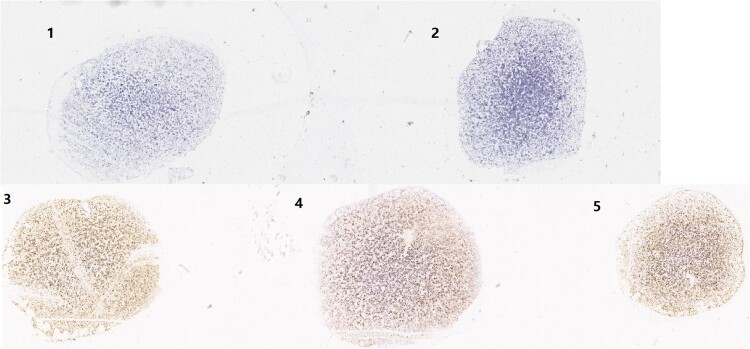
ScanScope images of the three HBoV2 mRNA transcripts at 72 h post-transfection. 1: HEK293 cells transfected with pBlueScript; 2: Untreated HEK293 cell control; 3: HEK293 cells transfected with pBlueScript-HBoV2 5043–5042; 4: HEK293 cells transfected with pBlueScript-HBoV2 5075–5074; 5: HEK293 cells transfected with pBlueScript-HBoV2 5220–5219.

To compare the quantities of the HBoV2 mRNA transcripts among the three HBoV2 recombinant plasmids, the RNAscope results were ScanScoped to visualize the images for dot-like nuclear signal calculation. Figure 5 shows the results for the cells at 72 h post-transfection. Spots labelled “pBlueScript vector” or “HEK293 cell” showed no dot-like nuclear signals. However, the spots labelled with pBlueScript-HBoV2 5043–5042, pBlueScript-HBoV2 5075–5074 and pBlueScript-HBoV2 5220–5219 showed very strong dot-like nuclear signals, especially those from pBlueScript-HBoV2 5075–5074.

### Immunofluorescence confocal microscopy and western blot protein analysis

**Figure 6.**
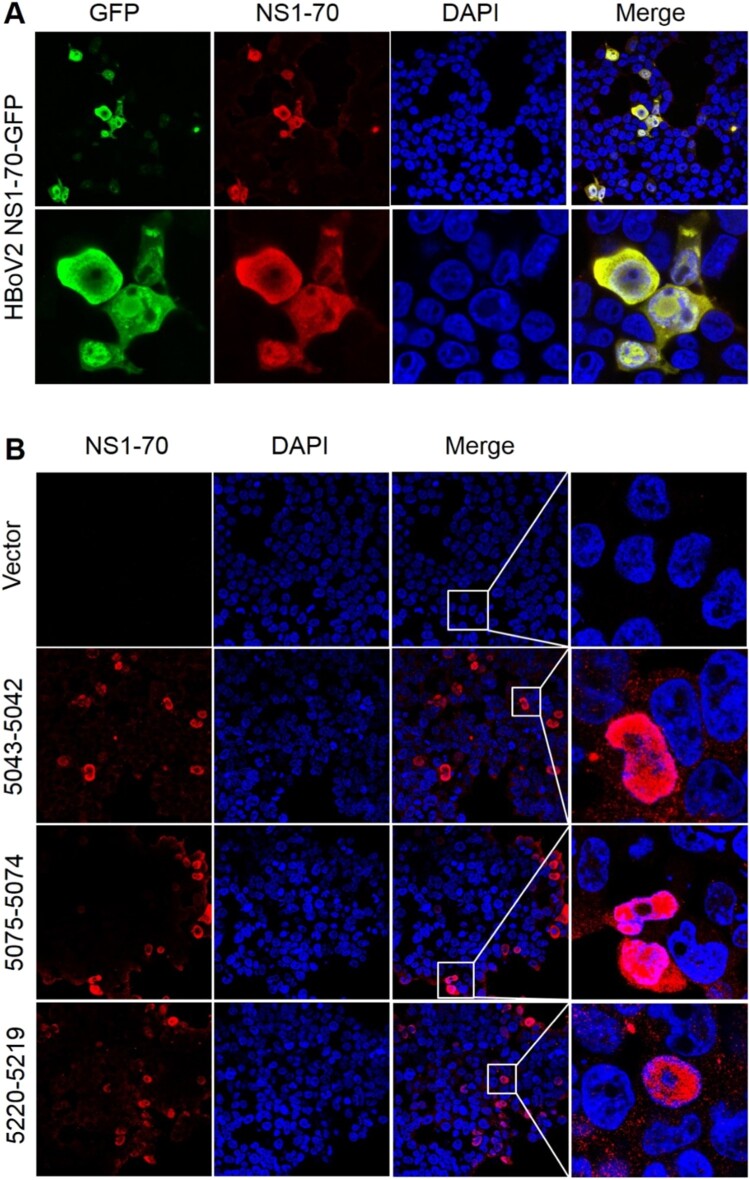
HBoV2 NS1-70 protein detection in HEK293 cells transfected with one of the three HBoV2 genomic recombinant plasmids by immunofluorescence confocal microscopy. A. HEK293 cells transfected with the HBoV2 NS1-70-GFP plasmid as the control were fixed and then stained with rabbit anti-HBoV2 NS1-70 at 72 h post-transfection (nuclei, blue; HBoV2 NS1-70-GFP, green; HBoV2 NS1-70, red). (B). HEK293 cells transfected with pBlueScript, pBlueScript-HBoV2 5043–5042, pBlueScript-HBoV2 5075–5074, and pBlueScript-HBoV2 5220–5219 plasmids were fixed and then stained with rabbit anti-HBoV2 NS1-70 at 72 h post-transfection (nuclei, blue; HBoV2 NS1-70, red). Insets show magnified views of the merged channels in the boxed regions. The images represent three independent experiments.

Using the recombinant HBoV2 NS1-70-GFP plasmid stained with HBoV2 NS1-70 rabbit polyclonal antibody as a positive marker (Figure 6A) to evaluate the efficiency of the HBoV2 NS1-70 rabbit polyclonal antibody, the expression of HBoV2 NS1-70 (red) was successfully detected under immunofluorescence confocal microscopy by this polyclonal antibody, and the GFP fluorescence signal (green) completely co-localized with the red fluorescence from NS1-70, which localized in both the cytosol and nucleus. These results show that the anti-HBoV2 NS1-70 polyclonal rabbit antibody specifically and efficiently recognized the NS1-70 protein from HBoV2.

The large amounts of protein (red) expressed in the cells separately transfected with the three recombinant plasmids co-localized with the nucleus (blue) in the DAPI stained nuclear region, with smaller amounts expressed in the cytosol at 72 h post-transfection (Figure 6B). HEK293 cells transfected with pBlueScript-HBoV2 5043–5042 reacted more strongly with the HBoV2 NS1-70 rabbit polyclonal antibody than those transfected with pBlueScript-HBoV2 5075–5074 or pBlueScript-HBoV2 5220–5219. However, there was no significant difference in the protein product quantities among the cells transfected with either of three recombinant plasmids.

**Figure 7.**
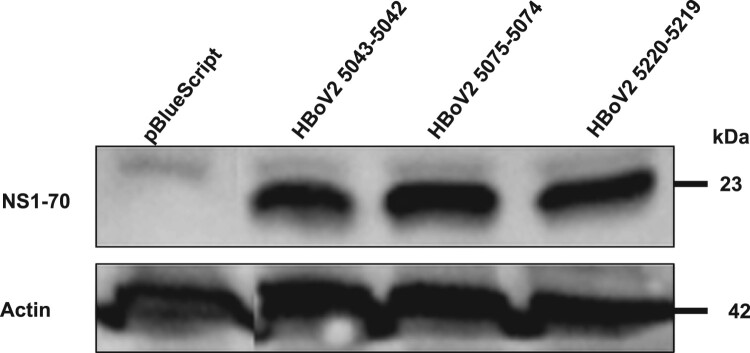
Western blot of HBoV2 NS1-70 protein expressed in HEK293 cells transfected with pBlueScript, pBlueScript-HBoV2 5043–5042, pBlueScript-HBoV2 5075–5074 and pBlueScript-HBoV2 5220–5219 plasmids at 72 h post-transfection. Cells lysates were analyzed by western blotting with HBoV2 NS1-70 rabbit polyclonal antibody or actin antibodies. Actin was used as the loading control.

The western blot in Figure 7 shows that after separately transfecting the HEK293 cells with the three different HBoV2 recombinant genomic plasmids they all expressed NS1-70 (molecular weight about ∼23 kDa), as confirmed by the same rabbit polyclonal antibody, and the protein product quantities did not differ significantly.

### Electron microscopically-observed virus particles

**Figure 8.**
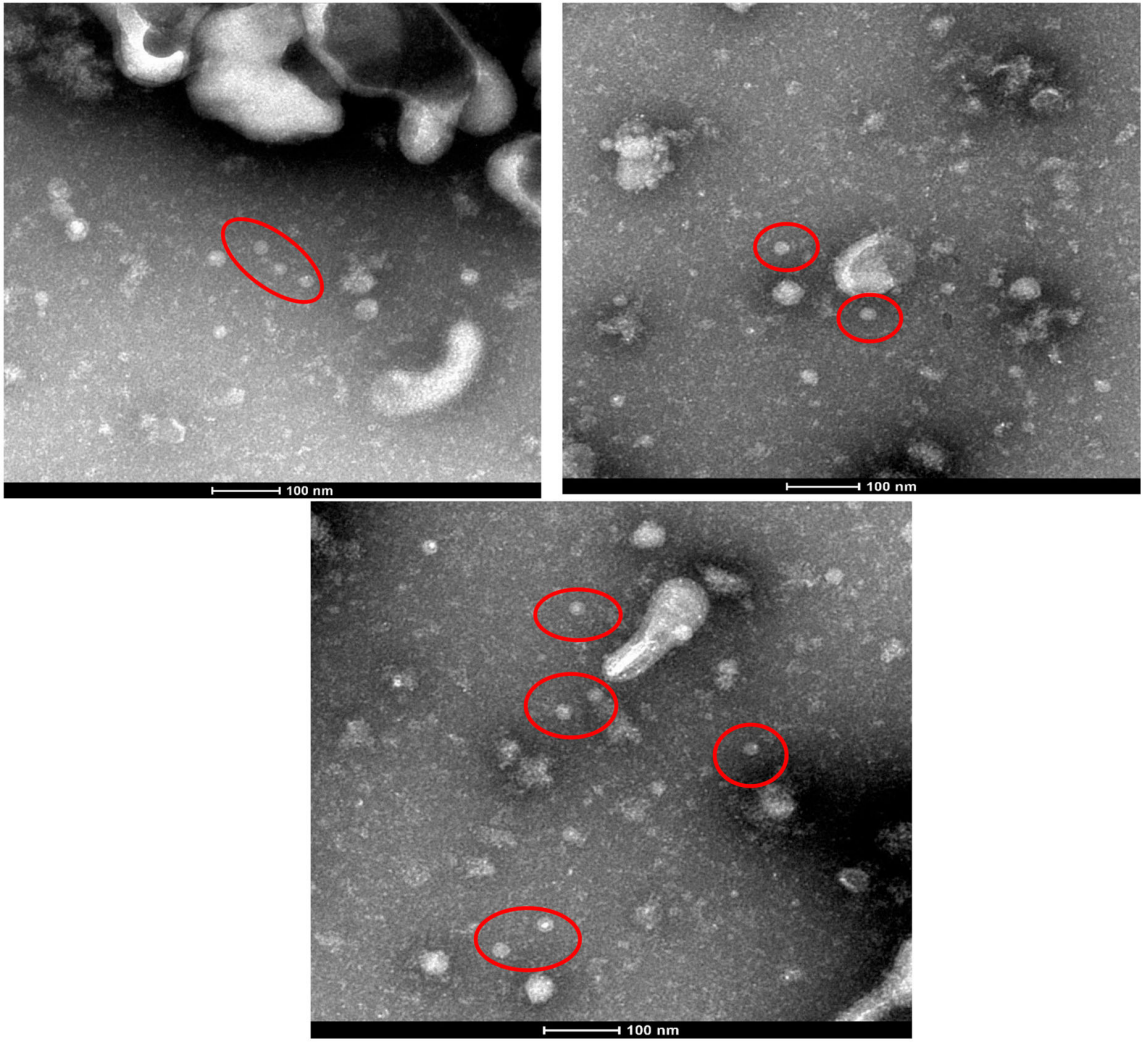
Red circles represent HBoV2 virus particles in HEK293 cells transfected with pBlueScript HBoV2 5043–5042, as detected by electron microscopy. The particles have the typical icosahedral appearance of parvoviruses, each with a diameter of approximately 20 nm.

At 72 h post-transfection in HEK293 cells transfected with either of the HBoV2 recombinant genomic plasmids or with the pBlueScript vector, HBoV virus particles were only visible in the cells transfected with pBlueScript HBoV2 5043–5042 (Figure 8).

When DNA from the pBlueScript-HBoV2 5043–5042-tranfected HEK293 cells was amplified with HBoV2 4F and HBoV2 4R primers, two bands were agarose gel electrophoresed: a slower migrating band of ∼5 kb (confirmed as the combined vector and partial HBoV2 sequences) and a faster migrating band of 1865bp (confirmed as the whole noncoding region sequence of HBoV2).

## Discussion

The circular 5307 nt HBoV2 (HBoV2-C1) genome, which is flanked by a 520 nt-long terminal NCR, was reported by our laboratory in 2012 [16]. Here, to reveal the role of HBoV2s circular genome, we constructed genomic clones to overcome the difficulty of not having appropriate cell lines for culturing HBoV2 virus or an experimental animal model for it [18], unlike HBoV1[19,20]. It has been assumed that the NCRs of HBoVs are likely to play important roles in bocavirus replication [14,21]. In the 520 nt-long terminal NCR of the circular HBoV2-C1 genome, three clusters of secondary structures exist: hairpins 1 and 2 and a 5′ terminal structure (Figure 3). Hence, we designed primers for nucleotide acid amplification to generate all three linearized HBoV2-C1 genomic clone constructs, including clones with all the secondary structures in the 5′ end of the genome (pBlueScript HBoV2 5043–5042), clones with both hairpins and the 5′ terminal structure at the 5′ end of the genome (pBlueScript-HBoV2 5075–5074), and clones with only the 5′ terminal structure at the 5’ end of the genome (pBlueScript-HBoV2 5220–5219).

By real-time PCR, dot-blot hybridization, ISH (RNAscope), immunofluorescence confocal microscopy and western blotting, we detected HBoV2-specific DNA, RNA and protein in the plasmid-transfected HEK293 cells.

rPCR revealed that HBoV2 gDNA levels in the recombinant plasmids increased in the HEK293 cells over time, as shown by the decreasing Ct values. The highest gDNA level occurred in cells transfected with pBlueScript-HBoV2 5075–5074 at 24 h post-transfection, whereas at 48 h and 72 h the levels were highest in the cells transfected with pBlueScript-HBoV2 5220–5219 and pBlueScript-HBoV2 5043–5042, respectively. However, the highest gDNA accumulation levels were in cells transfected with pBlueScript-HBoV2 5043–5042 at 72 h post-transfection, suggesting that retaining all secondary structures is important for DNA replication in HBoV2. It should be mentioned that the PCR not only detected signals from the viral DNA, but also the residual signals from the input plasmid. For replicated viral DNA, one has to use *Dpn*I to digest the input plasmids, and for progeny virions, one has to detect DNase I digestion-resistant viral DNA in the particles, as suggested by Huang et al. [10]. We attempted to discriminate DNA replication in the progeny virions generated from the transfected plasmids using *Dpn*I to digest the input plasmids. However, we failed to detect changes in the DNA content before and after *Dpn*I treatment (data not shown). Therefore, we chose not to determine the absolute DNA replication status of the generated progeny virions. We simply compared the relative changes in DNA quantity after separately transfecting the three different clonal plasmids into the cells to ascertain which clonal plasmid was the most effective one.

Dot-blot hybridization and RNAscope were used to detect HBoV2 mRNA in the cells, both of which had high sensitivity and specificity in the RNA test [22,23]. The highest multiple nuclear cytoplasmic signals from RNAscope and the dot-blot hybridization signals occurred in HEK293 cells transfected with pBlueScript-HBoV2 5075–5074, followed by pBlueScript-HBoV2 5220–5219 and pBlueScript-HBoV2 5043–5042. More mRNA was transcribed from pBlueScript-HBoV2 5075–5074 DNA, suggestive of a difference between replication and transcription in relying on the secondary structure of HBoV2 NCRs. The immunofluorescence confocal microscopy and western blot protein expression results showed that the three hetero-HBoV2 genomic clones were successfully constructed. These RNA and virus protein assays confirmed that the signals were produced from the viral DNA replication intermediates rescued from the transfected plasmids.

Based on the discovery of the extra-chromosomal circular episomal form of HBoVs, it is assumed that HBoVs establish latent infections in an episomal form, and these episomal structures may be the storage forms for persistent infection in human tissues [14]. However, in the present study, virus particles with a diameter of approximately 20 nm and the typical icosahedral appearance of parvoviruses were apparent only in HEK293 cells transfected with pBlueScript HBoV2 5043–5042, which contains all the NCR secondary structure of HBoV2 at the 5′ terminal, suggesting the importance of retaining all the secondary structure in the circular genome for virus assembly.

Our previous study found that the circular genome of HBoV2 was detectable in a faecal sample (BJQ435) [16], and had a low Ct value (Ct = 25.7) in rPCR, indicating the increased possibility of detecting the head-to-tail sequence in samples with higher viral loads [16]. A higher load for HBoV2 may indicate a higher risk of acute diarrhoea in children under 5 years old in Beijing [24]. Because DNA accumulation and virus particles were detected at the highest levels in HEK293 cells at 72 h post-transfection with plasmid pBlueScript HBoV2 5043–5042, which retains all the secondary structures, and the circular genome was detected for the same plasmid construct in HEK293 transfected cells, we hypothesized that the extra-chromosomal circular episomal form of HBoV2 plays important roles in DNA replication and virus assembly, and that the viral genome with all of its secondary structures present may be a marker for active infections with HBoVs. However, in-depth studies are needed to confirm this hypothesis.

## Conclusions

Recent data show that HBoV1 infection of human airway epithelium cultured at an air–liquid interface (HAE-ALI) induces a DNA damage response that facilitates viral genome amplification [25]. Other cell lines can also be infected with HBoV1 [7]. For instance, a HEK293-infectious HBoV1 clone has been established that can generate high titre progeny virions [19,26]. However, no cell line or experimental animal model has been found for HBoV2 propagation. In this study, we constructed three HBoV2 genomic clones that can replicate and transcribe mRNA in HEK293 cells. The circular nature of the HBoV2 genome was found to be dependent on the presence of all of the secondary NCR structures at its 5′ end, and these structures played important roles in DNA replication and virus assembly. Our findings support the rolling-cycle replication model of HBoVs.

## Ethics approval

This study was approved by the Ethics Committee of the Capital Institute of Pediatrics, Beijing, China.

## Supplementary Material

Supplemental Material
