# Supplementary material for: Keeping all secondary structures of the non-coding region in the circular genome of human bocavirus 2 is important for DNA replication and virus assembly, as revealed by three hetero-recombinant genomic clones
Source: Emerg Microbes Infect. 2019 Nov 1;8(1):1563–73. doi: 10.1080/22221751.2019.1682949 (PMC6832345; doi:10.1080/22221751.2019.1682949)
Supplement: Supplemental Material [file TEMI_A_1682949_SM3094.docx]

The supplementary materials:

***
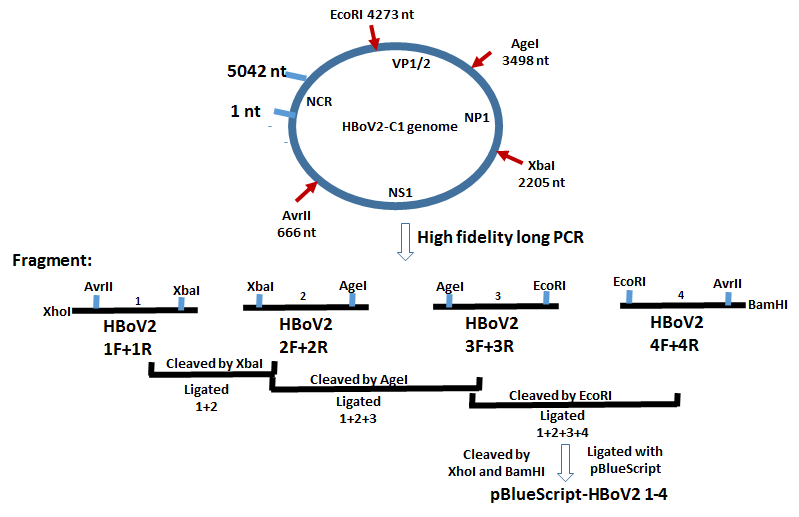
***

S1. The cloning scheme representation of the experimental strategy for clone construction of plasmid pBlueScript-HBoV2 1-4 containing the full length HBoV2 genome.


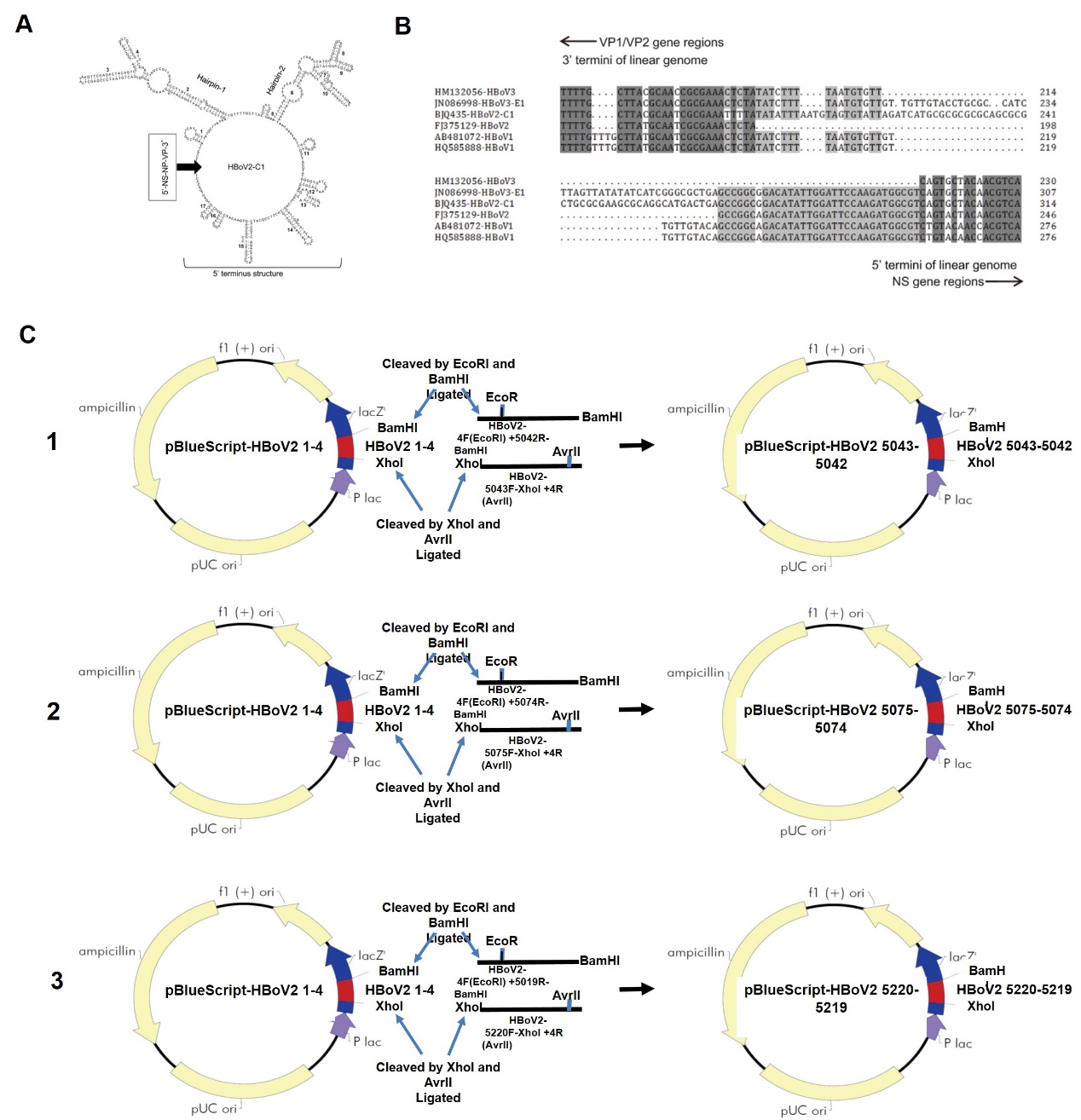


S2. Construction of three HBoV2 genomic recombinant plasmids

A: Secondary structure prediction and nucleotide alignment of the non-coding region (NCR) of HBoV2-C1 for the construction of three HBoV2 genomic recombinant plasmids. The arrow indicates the location of the region containing the NS, NP1, and VP1/VP2 genes (black rectangle), with the exception of the non-coding region, oriented 59 to 39. doi:10.1371/journal.pone.0048980.g003.

B: Alignment of known terminal genome of HBoV1, 2 and 3, in which the genome of HBoV2-C1 and JN086998-HBoV3-E1 is circular. The nucleotide number is counted according to the sequence of the non-coding regions by connecting 59 and 39 termini. doi: 10.1371/ journal.pone.0048980.g001.

C: Construction of three HBoV2 genomic recombinant plasmids named pBlueScript-HBoV2 5043-5042 (C-1), pBlueScript-HBoV2 5075-5074 (C-2) and pBlueScript-HBoV2 5220-5219 (C-3).
